# *Anastatus* Motschulsky (Hymenoptera, Eupelmidae): egg parasitoids of *Caligulajaponica* Moore (Lepidoptera, Saturniidae) in China

**DOI:** 10.3897/zookeys.881.34646

**Published:** 2019-10-17

**Authors:** Yong-Ming Chen, Gary A.P. Gibson, Ling-Fei Peng, Asim Iqbal, Lian-Sheng Zang

**Affiliations:** 1 Jilin Engineering Research Center of Resource Insects Industrialization/Institute of Biological Control, Jilin Agricultural University, Changchun, 130118, China Jilin Agricultural University Changchun China; 2 Honorary Research Associate, Agriculture and Agri-Food Canada, Canadian National Collection of Insects, Arachnids and Nematodes, K. W. Neatby Bldg., 960 Carling Avenue, K1A 0C6, Ottawa, Ontario, Canada Canadian National Collection of Insects Ottawa Canada; 3 State Key Laboratory of Ecological Pest Control for Fujian and Taiwan Crops, Fujian Provincial Key Laboratory of Insect Ecology, College of Plant Protection, Fujian Agriculture and Forestry University, Fuzhou, 350002, China Fujian Agriculture and Forestry University Fuzhou China

**Keywords:** *Antheraeapernyi*, Chalcidoidea, natural enemy, new species, parasitic wasp, taxonomy

## Abstract

Four species of *Anastatus* Motschulsky (Hymenoptera, Eupelmidae, Eupelminae) are newly reported as egg parasitoids of the Japanese giant silkworm, *Caligulajaponica* Moore and, as an alternate laboratory host, the Chinese oak silk moth, *Antheraeapernyi* (Guérin-Méneville) (Lepidoptera, Saturniidae) in China. The four species, *A.fulloi* Sheng & Wang, 1997, *A.gansuensis* Chen & Zang, **sp. nov.**, *A.japonicus* Ashmead, 1904, and *A.meilingensis* Sheng, 1998, were reared initially from eggs of *C.japonica* collected in Gansu, Jilin and Liaoning provinces and subsequently cultured in the laboratory on eggs of *A.pernyi*. An illustrated key to differentiate females of the four species, and males of some of the species is provided. Key features are illustrated, both sexes of the new species are described, and diagnoses of females of the other species are given.

## Introduction

The Japanese giant silkworm, *Caligulajaponica* Moore, 1862 (Lepidoptera, Saturniidae), is a widely distributed indigenous species in China, being reported previously from both Palaearctic (Heilongjiang, Jilin, Liaoning, Hebei, Shandong, Shaanxi) and Oriental (Chongqing, Fujian, Gansu, Guangdong, Guangxi, Guizhou, Hubei, Hunan, Jiangxi, Sichuan, Taiwan, Yunnan, Zhejiang) provinces (Qiao et al. 2014), as well as from Japan, North Korea and Russia (Li et al. 2009). Host plants include 38 species belonging to 30 genera in 20 families, including walnut (*Juglansregia* L., 1753), ginkgo (*Ginkgobiloba* L., 1771), chestnut (*Castaneamollissima* Bl., 1851), plum (*Prunus* spp. L., 1753), apple (*Maluspumila* Mill., 1768), sumac (*Toxicodendronvernicifluum* (Stokes) F. A. Barkley, 1937), pear (*Pyrus* spp. L., 1753) and persimmon (*Diospyroskaki* Thunb., 1780) (Yang et al. 2008; Liu and Feng 2013). In China, its most serious economic impact is as a defoliator of walnut and chestnut trees, causing millions of U.S. dollars of damage annually (Qiao et al. 2014). Currently, chemical pesticides are the most commonly used method to control this pest. Although some biological control techniques have been attempted, such as spraying the fungus *Beauveriabassiana* (Bals.-Criv.) Vuill., 1912, these have yielded unsatisfactory results (Liu and Luo 2008). Therefore, development of safe and effective methods for biological control of this pest is needed on an urgent basis.

Prior to the present study, seven species of Chalcidoidea (Hymenoptera) have been reported as parasitoids of *C.japonica* (sometimes cited under the combination *Dictyoplocajaponica*), including a single species of Chalcididae (*Kriechbaumerellahofferi* (Bouček, 1952)), another of Encyrtidae (*Ooencyrtusdictyoplocae* Sharkov, 1995) and Eulophidae (*Aprostocetusbrevipedicellus* Yang & Cao, 2015), two species of Eupelmidae (*Mesocomysalbitarsis* (Ashmead, 1904) and *M.kalinai* Ozidikmen, 2011) and of Trichogrammatidae (*Trichogrammachilonis* Ishii, 1941 and *T.dendrolimi* Matsumura, 1926) (Noyes 2019). Of these, *T.chilonis* and *T.dendrolimi* as well as a *Kriechbaumerella* sp. are also reported as parasitoids of the Chinese oak silk moth, *Antheraeapernyi* (Guérin-Méneville, 1855) (Lepidoptera, Saturniidae) (Noyes 2019).

Species of *Anastatus* Motschulsky (Eupelmidae, Eupelminae) are mostly primary parasitoids of the eggs of other insects, although a few have been reared as secondary parasitoids (hyperparasitoids) of Lepidoptera eggs through Scelioninae (Platygastroidea, Platygastridae) and Ichneumonidae (Ichneumonoidea) primary parasitoids, and some have been reared from Coleoptera larvae and Diptera puparia (Gibson 1995; Noyes 2019). Currently, about 150 valid species of *Anastatus* are described, making it the second largest genus of Eupelminae after *Eupelmus* Dalman (Noyes 2019). Seventeen described species are reported from China, all from the subgenus A. (Anastatus). Of these, Noyes (2019) listed 13 species from mainland China plus one other species (*A.formosanus* Crawford, 1913) separately from Taiwan. However, Noyes (2019) did not include *A.kashmirensis* Mathur, 1956, reported by Hu et al. (2011) or *A.acherontiae* Narayanan, Subba Rao & Ramachandra, 1960 and *A.gastropachae* Ashmead, 1904, reported by Yang et al. (2015), from mainland China. Although the *Anastatus*fauna of China has yet to be revised comprehensively, Yang et al. (2015) provided a key and imaged the females of *A.acherontiae*, *A.gastropachae*,and *A.japonicus* Ashmead, 1904, and Peng et al. (2017) provided an illustrated key to females of the six species (*A.dexingensis* Sheng & Wang, 1997, *A.flavipes* Sheng & Wang, 1997, *A.fulloi* Sheng & Wang, 1997, *A.shichengensis* Sheng & Wang, 1997, *A.huangi* Sheng & Yu, 1998, and *A.meilingensis* Sheng, 1998) described originally from China by Sheng and coauthors. Because of the extreme sexual dimorphism that characterizes Eupelminae (Gibson 1986, 1995), species recognition within the subfamily, including in *Anastatus*, has historically been based mostly on females and there is no key to the males that includes any Chinese species.

Several species of *Anastatus* have been used for biological control of various insect pests in China and elsewhere (Stahl et al. 2018), including *A.japonicus* in China against the litchi stink bug, *Tessaratomapapillosa* Stål, 1864 (Hemiptera, Tessaratomidae) (Li et al. 2014), and more recently *A.bifasciatus* (Geoffroy, 1785) in Europe against the invasive brown marmorated stink bug, *Halyomorphahalys* Stål, 1855 (Hemiptera, Pentatomidae) (Hancock et al. 2018; Judith et al. 2018). As part of studies to discover potentially new biological control agents of *C.japonica* in China, members of the Institute of Biological Control, Jilin Agricultural University, collected eggs of this species in Gansu, Jilin and Liaoning provinces and reared these in the laboratory for parasitoids. This resulted in what we provisionally identify as two species of *Mesocomys* Cameron (Eupelmidae, Eupelminae), four species of *Anastatus*, and one species of *Aprostocetus* Westwood (Eulophidae, Tetrastichinae). Here we report on the species of *Anastatus* reared from *C.japonica* eggs, which were cultured subsequently in the laboratory on the eggs of an alternate host, *A.pernyi*. Based on females reared in our surveys we differentiated four morphospecies of *Anastatus* that we identify as three previously described species, *A.fulloi*, *A.japonicus* and *A.meilingensis*, plus one species new to science. Here we describe that new species. The culturing of the other three described species also provided fresh specimens of both sexes. We therefore present an illustrated key to both sexes of the four species of *Anastatus* reared as egg parasitoids of *C.japonica*, except for the males of *A.fulloi* and *A.japonicus* that we cannot reliably differentiate.

## Material and methods

### Surveys and rearing

Surveys and rearing of specimens on which this study is based were conducted by Yong-Ming Chen, Lian-Sheng Zang and Asim Iqbal. Surveys were conducted in three provinces in China that were infested with *C.japonica*, one province in north-central China (Gansu, Kangxian County, 33°26'33.81"N, 105°41'52.10"E in January to March, 2017 and 2018) and two provinces in north-eastern China (Jilin, Changchun City, 43°48'29.09"N, 125°24'14.03"E in July to August, 2017, and Liaoning, Benxi County, 41°14'56.40"N, 124°28'55.06"E in March, 2017). The overwintering egg masses collected in the field surveys were placed into glass tubes and the opening of each tube covered with a small piece of cloth. The glass tubes were kept in a rearing chamber kept 25±1° C and 70±5% RH, with a 14 L: 10 D photoperiod until the wasps emerged. The sex ratio = was calculated as the number of females/(number of females + number of males). Emerging wasps were then used to establish colonies at Jilin Agricultural University using fresh and healthy unfertilized eggs of *A.pernyi* that were obtained by dissecting female abdomens as per Li et al. (2018).

### Taxonomy

Random samples of reared colony individuals were preserved in 75% ethanol for further use, with specimens used for morphological study and collection deposition either air dried and point-mounted at JLAU or critical-point dried from ethanol and point-mounted at CNC. Species identifications were made using the keys and illustrations provided by Kalina (1981), Narendran (2009), Yang et al. (2015) and Peng et al. (2017), plus examination of type and other identified material in CNC, FAFU and USNM. The description of the new species is based on specimens reared in the laboratory from the eggs of *A.pernyi* that were observed and measured in micrometers using a Keyence VHX-2000 digital microscope at JLAU or with a Nikon SMZ 1500 binocular microscope with an ocular grid having 100 divisions at the CNC. Macrophotographs and plates of illustrations of all treated species were made at the CNC using a Leica DMC5400 20-megapixel camera attached to a Leica Z16 APO motorized macroscope. A Leica, 100-watt halogen light source was used to illuminate specimens for the descriptions whereas three Leica KL2500 LCD fiber optic light sources fitted with 250-watt cold light reflector lamps were used to photograph the specimens. To reduce glare, the halogen light source was filtered through a piece of translucent Mylar tracing acetate taped to the microscope objective, whereas the fiber optic light sources were filtered through a polystyrene foam dome. Because of this, color illustrated in the plates of illustrations may differ somewhat from the described color. Serial images were combined with Zerene Stacker and digitally retouched as necessary using Adobe Photoshop to enhance clarity. Imaged specimens bear a unique ‘CNC Photo 2019-×’ number label, of which “×” is cited between parentheses in the figure captions to indicate the specimen imaged. All imaged specimens are in the Canadian National Collection of Insects and Arachnids.

Morphological and sculptural terms follow Gibson (1995, 1997, 2011) and Gibson and Fusu (2016). Attributes of both sexes of the new species are described under four separate headings-color, setation, structure, and sculpture, except female fore wing color and setal patterns are described together because the features are strongly correlated. For descriptive purposes, the terms ‘dark’ and ‘pale’ are used as general terms relative to one another, with dark referring from dark brown to black, often also with variably conspicuous metallic lusters, and pale referring to light brown to orange or yellow. The fore wing is subdivided into two regions, the ‘basal’ and ‘discal’ regions. The basal region of a macropterous individual is the region basal to the basal fold behind the costal cell whereas the discal region is apical to that fold; in brachypterous females, the basal region is the more hyaline and less setose basal region and the discal region is the more densely setose apical region. Interpretation of the folds and regions of the basal region of the fore wing follows Gibson (2004). Abbreviations used in the text or plates of figures are: **clv** = clava, **cbr** = remnant of hyaline cross band of fore wing, **flx** = flagellomere 1–8, **Gtx** = gastral tergite 1–6, **LOL** = minimum distance between anterior and posterior ocellus, **MPOD** = maximum diameter of posterior ocellus, **mps** = multiporous plate sensilla, **MV** = marginal vein, **OOL** = minimum distance between posterior ocellus and inner orbit, **POL** = minimum distance between posterior ocelli, **pdl** = pedicel, **PMV** = postmarginal vein, **STV** = stigmal vein, **syn** = syntergum. Measurement of scape length does not include the radicle. Width of antennal segments does not include the length of projecting setae (Gibson 1989, 2011). Total body length excludes the ovipositor. Type material of the new species and voucher specimens of the other three treated species of *Anastatus* have been deposited in the following institutions as listed in the species treatments:

**AICF** Al. I. Cuza University, Iaşi, Romania, Lucian Fusu collection;

**BMNH**The Natural History Museum, Department of Entomology, London, England;

**CNC**Canadian National Collection of Insects, Arachnids and Nematodes, Agriculture and Agri-Food Canada, Ottawa, ON, Canada;

**FAFU** Biological Control Research Institute, Fujian Agriculture and Forestry University, Fuzhou, China;

**JLAU** Institute of Biological Control, Jilin Agriculture University, Changchun, China;

**IZCAS**Institute of Zoology, Chinese Academy of Sciences, Beijing, China;

**USNM**National Insect Collection, National Museum of Natural History, Smithsonian Institution, Washington, DC, USA.

## Results

### Survey and rearing

From the cultures established from the surveys in Gansu Province, seven species were ultimately identified as egg parasitoids of *C.japonica*, belonging to two families and three genera (*Anastatus* (4 spp.) and *Mesocomys* (2 spp.) and *Aprostocetus* (1 sp.)). In 2017, three species were reared, *A.gansuensis* sp. nov., *M.albitarsis* (Ashmead) and *M.trabalae* Yao, Yang & Zhao, 2009. In 2018, all seven species were reared and identified as *A.fulloi*, *A.gansuensis*, *A.japonicus*, *A.meilingensis*, *M.albitarsis*, *M.trabalae*, and *Aprostocetusbrevipedicellus*. The proportion of females to males under natural field conditions was 0.75, 1.00, 0.89, 0.75, 0.66, 0.66, and 0.77, respectively, for the seven species. In 2017 in Jilin Province only *A.japonicus* was reared, whereas in Liaoning province *A.fulloi* and *M.albitarsis* were reared. Their sex ratios were 0.82, 0.73, and 0.68, respectively. All seven species were reared successfully on the eggs of the alternative host, *Antheraeapernyi*, under laboratory conditions.

### Systematics

#### Anastatus (Anastatus)

Taxon classificationAnimaliaHymenopteraEupelmidae

Motschulsky

8449582F-C3B7-5DCB-80CC-7A749452F920

Anastatus Motschulsky, 1859: 116. Type species: Anastatusmantoidae Motschulsky, by monotypy.
Anastatus (Anastatus) ; Gibson 1995: 105, 111.

##### Remarks.

For a complete listing of the extensive generic synonymy of *Anastatus* see Gibson (1995) and Noyes (2019). Bouček (1977) recognized two subgenera in *Anastatus*, the nominate subgenus and A. (Cladanastatus) Bouček, 1979). Currently, the nominate subgenus comprises all presently described species except for the type species of A. (Cladanastatus). Females and males of *Anastatus* and A. (Anastatus) can be distinguished from those of other genera of Eupelminae using the key by Gibson (1995).

### Key to species of A. (Anastatus) reared from *Caligulajaponica* eggs in China

**Table:** 

1	Female	**1**
–	Male	**5**
2(1)	Brachypterous, fore wings extending only to about posterior margin of Gt1 (Fig. 6A–E), with discal region densely setose with slightly lanceolate dark setae except for distinct region of orangish setae medially behind marginal vein and often a very slender, sometimes interrupted remnant of a hyaline cross band distally (Fig. 6E: cbr)	***A.meilingensis* Sheng**
–	Macropterous, fore wings extending to apex of gaster (Figs 1A, 2B, 5A) and discal region with broad hyaline cross band with white setae extending across wing behind marginal vein (Figs 1E, 3A, 5E)	**3**
3(2)	Acropleuron often dark anteriorly to about level of base of fore wing but variably paler, light brown to orangish or yellowish over at least posterior half (Fig. 5D) in contrast to dark mesonotum (Fig. 5C)	***A.japonicus* Ashmead**
–	Acropleuron entirely dark and not contrasting in color with mesonotum or at most somewhat paler anteriorly only near prepectus, the paler region not nearly extending to level of base of fore wing (Figs 1D, 2F)	**4**
4(3)	Procoxa pale, orangish to yellowish (Fig. 2F), similar in color to lateral surface of pronotum but much lighter than posternum; concave part of mesoscutal medial lobe medially setose with white setae for width only about equal to width of bare region on either side (Fig. 2E); mesotarsus, excluding tarsal pegs, uniformly pale (Fig. 3E); fore wing hyaline cross band with apical margin more strongly V-like angulate than basal margin (Fig. 3A) and usually with a few isolated dark setae medially within hyaline band (Fig. 3C)	***A.gansuensis* Chen & Zang, sp. nov.**
–	Procoxa dark, much darker than lateral surface of pronotum and similar in color to prosternum (Fig. 1D); concave part mesoscutal medial lobe setose with white setae for almost entire width (Fig. 1C); mesotarsus with basal two tarsomeres obviously darker than more apical tarsomeres over at least dorsal and posterior surfaces (Fig. 3F, G); fore wing hyaline cross band with apical and basal margins similarly curved and without isolated dark setae within band (Fig. 1E)	***A.fulloi* Sheng & Wang**
5(1)	Clava at least as long as combined length of previous three funiculars (Figs 1H, 5H)	***A.fulloi* Sheng & Wang and *A.japonicus* Ashmead**
–	Clava shorter than combined length of previous three funiculars (Figs 4H, 6H)	**6**
6(5)	Metatibia mostly similarly dark as femur, pale basally for distance only about equal to own apical width or about one-quarter or less length of tibia (Fig. 4B)	***A.gansuensis* Chen & Zang, sp. nov.**
–	Metatibia with about basal half pale and apical half darker, though lighter brown than femur (Fig. 6F)	***A.meilingensis* Sheng**

### Species treatments

#### Anastatus (Anastatus) fulloi

Taxon classificationAnimaliaHymenopteraEupelmidae

Sheng & Wang

D86E2606-81DD-506E-B2D5-F81D4E041EAF

Fig. 1A–H

Anastatus fulloi Sheng & Wang in Sheng et al., 1997: 61–62, figs 14, 15; holotype (JLAU), examined.
Anastatus fulloi ; Peng et al. 2017: 10–13, figs 19–27.

##### Diagnosis.

**Figure 1.**
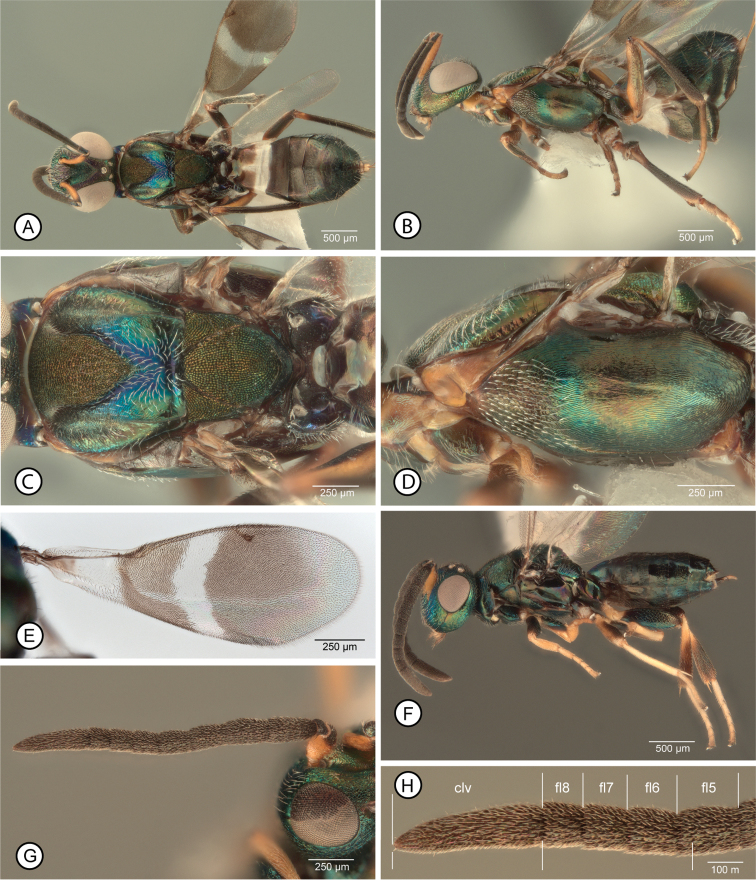
*Anastatusfulloi***A–E** female: **A** dorsal habitus (13) **B** lateral habitus (12) **C** dorsal mesosoma (13) **D** lateral mesosoma (12) **E** fore wing (14). **F–H** male: **F** lateral habitus (22) **G** antenna (32) **H** clava and apical three funiculars (32) (three lower bars indicate length of clava compared to combined length of apical funiculars). Abbreviations: clv = clava, flx = flagellomere number.

**Female.** Macropterous (Fig. 1A); fore wing with broad hyaline cross band behind marginal vein with similarly curved basal and apical margins so band uniformly wide, and without isolated dark setae medially (Fig. 1E). Mesosoma (excluding legs) dark except prepectus and lateral, vertical surface of pronotum entirely or mostly pale (Fig. 1A–D); procoxa dark, similar in color to prosternum and acropleuron (Fig. 1D). Mesoscutum with posterior concave portion almost entire setose (Fig. 1C). Mesotarsus with basal two tarsomeres infuscate over at least dorsal and posterior surfaces, obviously darker than subsequent two tarsomeres (Fig. 3F, G). Antenna with at least apical funicular slightly transverse and previous one or two funiculars subquadrate (Fig. 1B).

**Male.** Structure plus color, setal and sculptural patterns (Fig. 1F) similar to those described for *A.gansuensis* except clava at least about as long as combined length of fl6–fl8 + 0.25 apical length of fl5 (Fig. 1G, H), and sometimes as long as combined length of fl5–fl8, with fl8 quadrate to slightly transverse, fl7 quadrate to slightly longer than wide, and fl6 and fl5 longer than wide (Fig. 1H).

##### Distribution.

*Anastatusfulloi* (Genbank accession no. MK604241) was described originally from Jiangxi Province from two localities (Wuyi Mountains and Meiling) (Sheng et al. 1997) as detailed by Peng et al. (1997). We reared it in the field from the following two new localities: **Gansu Province**: Longnan City, collected 23.I.2018, Yong-Ming Chen (1♀, 1♂ AICF; 1♀, 1♂ BMNH; 19♀, 21♂ CNC; 5♀, 3♂ FAFU; 12♀,7♂ IZCAS; 1♀, 1♂ USNM). **Liaoning Province**: Benxi, Manchu Autonomous Co., Benxi City, Tai Shan Forest Farm, 23.IV.2017, Yong-Ming Chen.

##### Hosts.

The type series was reared from the eggs of the yellow spotted stink bug, *Erthesinafullo* (Thunberg, 1783) (Hemiptera, Pentatomidae). Here we newly report it as an egg parasitoid of the lepidopteran hosts *C.japonica* and, in the laboratory, *A.pernyi*.

##### Remarks.

Of the species we reared from *C.japonica*, females of *A.fulloi* are most similar to those of *A.gansuensis* because they are fully winged (cf. *A.meilingensis*) and have a dark acropleuron that does not contrast with the mesonotum (cf. *A.japonicus*). However, females differ from those of *A.fulloi* in color, setal and structural features, as given in the key to species and diagnoses.

Sheng et al. (1997, fig. 14) and Peng et al. (2017, figs 25, 26) described and illustrated the flagellar structure of males from the type series of *A.fulloi* reared from *E.fullo*; they have the clava at least as long as the previous four segments combined. However, the males we reared with females from *A.pernyi* eggs that we identify as *A.fulloi* have the clava quite obviously shorter than the combined length of the four preceding funiculars (Figs 1G, H), being about as long as the combined length of fl6–fl8 plus about the apical quarter (Fig. 1H) to half of fl5. The difference between the flagellar structures observed between our males and those of the type series could be explained by populational or host correlated differences or, perhaps, indicate two cryptic species that are more host-taxon restricted than is currently considered for *A.fulloi*. Regardless, compared to males of *A.gansuensis* (Fig. 4H) and *A.meilingensis* (Fig. 6H), males of *A.fulloi* (Fig. 1H) have somewhat short apical funiculars so that the clava relative to the combined length of the funiculars is greater. Because of similarity in their flagellar structures (cf. Figs 1H, 5H) we cannot currently reliably differentiate *A.fulloi* from *A.japonicus* males.

#### Anastatus (Anastatus) gansuensis

Taxon classificationAnimaliaHymenopteraEupelmidae

Chen & Zang sp. nov.

A1C7FBB8-3814-56EF-A36D-92EB77F4B0F6

http://zoobank.org/B1EC8F11-A663-4F1B-B6E2-189224691C5F

**Figure 2.**
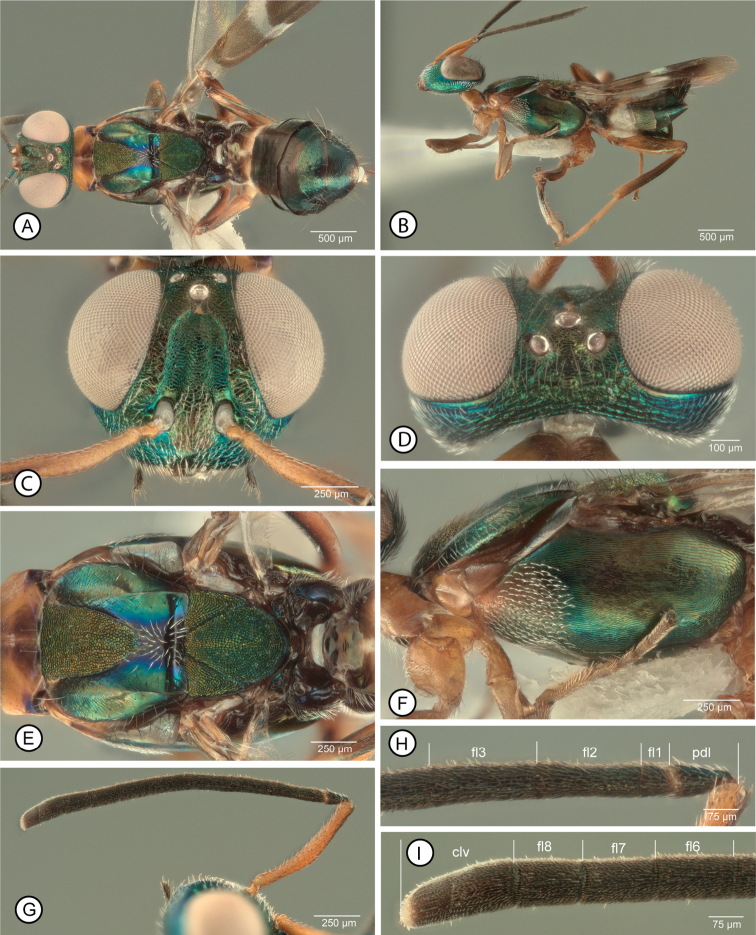
*Anastatusgansuensis*, female **A** dorsal habitus (9) **B** lateral habitus (7) **C** head in frontal view (10) **D**, same in dorsal view (9) **E** mesosoma in dorsal view (9) **F** same in lateral view (7) **G** antenna (10) **H** pedicel and basal three funiculars (10) **I** clava and apical three funiculars (10). Abbreviations: clv = clava, flx = flagellomere number; pdl = pedicel

**Figure 3.**
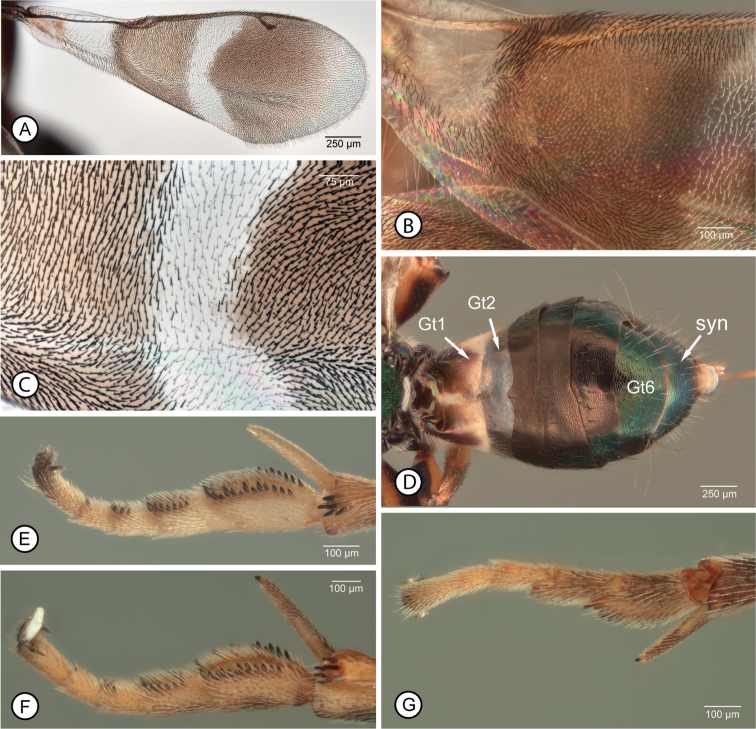
*Anastatus* spp., female **A–E***A.gansuensis*: **A** fore wing (8) **B** basal half of fore wing (29) **C** enlargement of medial part of wing disc (8) **D** gaster in dorsal view (9) **E** mesotarsus and apex of mesotibia, in anterior view (7). **F, G***A.fulloi*, mesotarsus and apex of mesotibia (12): **F** in anterior view **G** in posterodorsal view. Abbreviations: Gtx = gastral tergite number, syn = syntergum.

**Figure 4.**
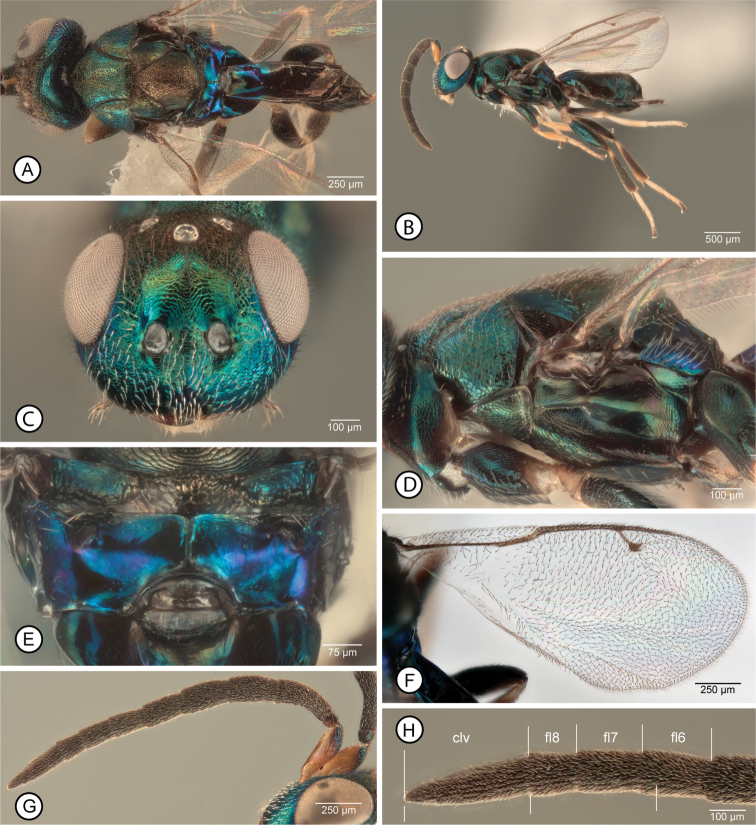
*Anastatusgansuensis*, male **A** dorsal habitus (17) **B** lateral habitus (16) **C** head in frontal view (19) **D** mesosoma in lateral view (16) **E** propodeum (17) **F** fore wing (17) **G** antenna (18) **H** pedicel and basal three funiculars (18) (three lower bars indicate length of clava compared to combined length of apical funiculars). Abbreviations: clv = clava, flx = flagellomere number.

Figs 2A–I , 3A–G , 4A–H

##### Type material.

***Holotype*** ♀ (FAFU), Shanchacun, Yuntai Town, Kangxian County, Gansu Province, China, 01.III.2017, Yong-Ming Chen. ***Allotype*** ♂ (FAFU), same data as holotype (from subsequently established colony on *A.pernyi*).

##### Additional paratypes.

Same data as holotype (2♀, 1♂ FAFU). Gansu Province, Kang Co., Longnan City, collected 23.I.2018, Yong-Ming Chen, ex. *Caligulajaponica* Moore egg, lab reared on eggs of *Antheraeapernyi* (Guérin-Méneville) (Lep: Saturniidae) (2♀, 1♂ AICF; 2♀, 1♂ BMNH; 67♀, 18♂ CNC; 5♀, 3♂ FAFU; 6♀ IZCAS; 2♀, 1♂ USNM).

##### Diagnosis.

**Female.** Macropterous (Fig. 2B); fore wing with broad hyaline cross band behind marginal vein and with apical margin more distinctly angulate (V-like) than basal margin such that length of hyaline band along marginal vein about twice medial length of hyaline band (Fig. 3A), and usually with a few isolated dark setae within band medially (Fig. 3C). Mesosoma (excluding legs) dark except prepectus and pronotum or at least lateral, vertical surface of pronotum, pale (Fig. 2A, B, E, F); procoxa pale, similar in color to lateral surface of pronotum (Fig. 2F). Mesoscutum with posterior concave portion setose only medially, width of setose region about equal to width of bare region on either side (Fig. 2E). Mesotarsus (excluding tarsal pegs) with at least basal four tarsomeres similarly pale (Fig. 3C). Antenna with all funiculars (fl2–fl8) longer than wide (Fig. 2G–I).

**Male.** Legs with all femora mostly to entirely dark, mesotibia entirely pale, and metatibia mostly dark, pale basally for a distance at most equal to about apical width or about one-quarter length of tibia (Fig. 4B). Flagellum (Fig. 4G) with clava distinctly shorter than combined length of fl6–fl8, with at least fl6 and fl7 obviously longer than wide (Fig. 4H).

##### Description.

**Female** (habitus, Fig. 2A, B). Body length 2.2–3.9 mm.

***Color.*** Head (Fig. 2C, D) mostly metallic green to bluish-green but with variably extensive and distinct coppery to reddish-violaceous lusters on at least parascrobal region and interantennal prominence (Fig. 2C) and sometimes on frontovertex, including ocellar triangle (Fig. 2D). Maxillary and labial palpi dark brown. Antenna with scape mostly pale, yellowish (Fig. 2G), except variably extensively darker brown and sometimes with slight metallic luster dorsoapically (Fig. 2C); pedicel and flagellum dark brown (Fig. 2G–I).

Pronotum mostly pale (Fig. 2E, F) except black posterolaterally anterior each spiracle, and concave, dorsomedial part often variably darker brownish (Fig. 2A, E); propleuron similarly pale as lateral surface of pronotum but prosternum dark. Mesonotum (Fig. 2E) mostly dark with metallic green to bluish-green lusters similar to head except convex part of mesoscutal medial lobe and/or scutellar-axillar complex anteriorly usually with slight coppery to reddish lusters, and concave posteromedial part of mesoscutum more distinctly blue to purple adjacent to medially dark violaceous to black setose part (Fig. 2E). Prepectus similarly pale as lateral surface of pronotum (Fig. 2F). Tegula dark brown. Mesopleurosternum mostly dark with greenish luster except variably distinctly paler anterodorsally near prepectus, the paler region at most extending to or slightly beyond level of base of tegula, and strigose medial part of acropleuron usually with slight coppery to reddish-violaceous lusters under some angles of light (Fig. 2F). Front leg with coxa, trochanter and trochantellus similarly pale as lateral surface of pronotum and prepectus; femur with anterior and/or dorsal surfaces and tibia with anterior surface similarly pale as coxa, but otherwise variably dark brown; tarsus with at least apical tarsomere brown but basal four tarsomeres pale to variably dark brown. Middle leg with trochanter and trochantellus at least ventrally dark; femur with dorsal surface longitudinally dark at least posteriorly, but ventral surface and usually dorsal surface anteriorly paler, and more whitish-translucent, anteroapically; tibia dark dorsally but paler basally, ventrally, and apically except for dark apical pegs; mesotibial spur and mesotarsus similarly pale, yellowish-white to white, except for dark pegs (Fig. 3E). Hind leg with at least ventral surface of trochanter and trochantellus dark; femur with dorsal and ventral surfaces paler than longitudinally dark anterior and posterior surfaces; tibia with dorsal surface dark but paler basally, apically and ventrally; tarsus with at least apical tarsomere brown and often one or two basal tarsomeres variably distinctly brownish. Macropterous, with fore wing extending beyond gastral apex when folded over dorsum (Fig. 2B); fore wing (Fig. 3A) with basal region mostly hyaline but brownish basally, with dark setae posteriorly along vanal and cubital areas and apically within basal brownish part (Fig. 3B), and hyaline part with whitish-translucent setae over at least about posterior half bare but behind length of SMV from level of base of parastigma; discal region (Fig. 3A) brownish from base of parastigma to level slightly beyond apex of PMV but hyaline towards wing apex and with complete hyaline cross band behind MV; hyaline cross band with white hair-like setae except for a few isolated dark setae within band medially (Fig. 3C), with apical margin reaching base of STV along leading margin and noticeably angulate compared to more evenly curved basal margin such that length of hyaline band along MV is about twice medial length (Fig. 3A); brownish region basal to hyaline cross band with slightly lanceolate dark brown setae except often for variably distinct, more orangish region of lanceolate setae behind parastigma and base of MV anterior to medial fold (when apparent, orangish region of setae reaching parastigma and base of MV, but at least narrowly separated by dark brown setae from basal cell basally and hyaline cross vein apically, Fig. 3B), and with entirely dark setae beyond hyaline cross band, the setae slightly lanceolate behind venation but more hair-like apically within more hyaline part. Metanotum and propodeum (Fig. 2E) dark brown or with slight metallic purple luster.

Gaster (Fig. 3D) mostly dark but syntergal flange and ovipositor sheaths pale, yellowish; in dorsal view Gt1 brown basally and variably extensively white apically and Gt2 translucent-hyaline so with sub-basal whitish region (Fig. 3D), but in lateral view basal two sternites entirely white (Fig. 2B); Gt3–Gt5 dark brown or with slight violaceous luster, and Gt6 and syntergum except for flange with green to bluish luster (Fig. 3D).

***Setation.*** Head with slightly lanceolate, whitish-translucent setae on lower face, interantennal prominence, lower parascrobal region (Fig. 2C) and gena behind eye (Fig. 2B, D), and with dark hair-like setae on upper parascrobal region and frontovertex (Fig. 2D).

Pronotum mostly bare but with a few long, bristle-like setae in transverse line anterior to spiracle and similar seta medially on either side of mediolongitudinal groove (Fig. 2E). Mesonotum (Fig. 2E) with convex anteromedial mesoscutal lobe, uniformly, inconspicuously setose with whitish hair-like setae, outer convex surface of lateral lobe laterally with 1–2 rows of somewhat longer whitish setae, and posteromedial concave part of mesoscutum with more conspicuous, very slightly lanceolate white setae medially, the region of white setae about as wide as bare region on either side of band and not extending to line of dark hair-like setae on inner inclined surface of lateral lobe near dorsally carinate posteromedial part of lateral lobe; scutellar-axillar complex bare, except for dark setae laterally on axillae and posterolaterally on scutellum, the posteriormost setae somewhat longer and more bristle-like than others. Mesopleurosternum sparsely setose with whitish-translucent hair-like setae ventrally and more densely, uniformly setose with very slightly lanceolate whitish-translucent setae anterolaterally to level about equal with apex of tegula; acropleuron bare (Fig. 2F). Propodeum bare except for a few slightly lanceolate white setae along anterior margin lateral of spiracle and along extreme lateral margin (Fig. 2E).

***Structure.*** Head in frontal view (Fig. 2C) about 1.3× wider than high; in dorsal view (Fig. 2D) width about 2× length and about 3.5× minimum distance between eyes, with vertex gradually curved to occiput without margin or carina; in lateral view about 1.6× higher than long; eye height about 2× length of malar space; OOL: LOL: POL: MPOD (holotype) = 3: 6: 11.4: 6.2; scrobal depression (Fig. 1C) with abrupt to carinate margins laterally but dorsally margins less distinct and not complete below anterior ocellus, separated medially by short, lighter colored line or vertical ridge below ocellus, with indistinctly delimited dorsal margin separated from anterior ocellus by distance slightly less than longitudinal diameter of ocellus. Antenna (holotype) with relative length (width) of scape = 43(7); pedicel = 10(5); fl1–fl8 = 4(5), 13(6), 15(6), 16(7), 14(7), 14(7.5), 12(8), 11(8); clava 23(9).

Mesoscutum (Fig. 2E) slightly wider than long, with anterior, convex part of medial lobe V-shaped with slightly sinuate to slightly outcurved sides convergent posteriorly for about 0.7× medial length of mesoscutum; lateral lobe carinately margined over almost posterior half; medial length of scutellar-axillar complex, including frenal area, about as long as maximum width with scutellum about 1.25× as long as own width, Fore wing about 2.7× as long as maximum width; smv: mv: pmv: stv (holotype) = 51: 70: 33: 10. Propodeum typical of genus, bowtie-like (Fig. 2E). Profemur with ventral margin evenly arched, without abrupt angulation or denticle within about apical third, and with 2 or 3 dorsoapical pegs. Mesotibia with apical patch of 3–6 pegs (Fig. 3E). Mesotarsus with single row of pegs on either side of basal three tarsomeres and single peg apically on either side of penultimate tarsomere (Fig. 3E).

Gaster with ovipositor sheaths at least slightly protruding beyond syntergal flange (Fig. 3D), but for distance up to about 2× length of flange.

***Sculpture.*** Head with vertex and interocellar triangle transversely alutaceous to alutaceous-strigose, upper parascrobal region and frons to posterior ocelli on either side of anterior ocellus more mesh-like coriaceous to coriaceous-imbricate or coriaceous-granular, and lower half of parascrobal region reticulate-rugulose; lower face reticulate to reticulate-rugose; interantennal prominence reticulate-imbricate; scrobal depression mostly reticulate-imbricate.

Mesoscutum with convex anterior part of medial lobe uniformly punctate-reticulate, posteromedial concave part or medial lobe smooth and shiny, and lateral lobes mostly finely mesh-like coriaceous to alutaceous, with sculpture of inner inclined surfaces more distinct lateral of anteromedial lobe and much finer to smooth and shiny posteriorly near scutellar-axillar complex. Scutellar-axillar complex uniformly, similarly punctate-reticulate as convex part of medial lobe. Mesopleurosternum with anterolateral region finely mesh-like coriaceous but dorsally bare part near tegula more alutaceous-coriaceous; acropleuron mostly longitudinally strigose, more minutely centrally, sculpture dorsally and ventrally more longitudinally strigose-coriaceous and posteriorly mesh-like coriaceous with larger cell size.

Gaster (Fig. 3D) with Gt1 and Gt2 dorsally almost smooth, only obscurely and very finely mesh-like coriaceous, Gt3–Gt5 more distinctly, somewhat transversely, coriaceous-reticulate with sculpture defined by slightly raised lines, and Gt6 and syntergum more transversely coriaceous-alutaceous to mesh-like coriaceous.

**Male** (habitus, Fig. 4A, B). Body length 2.0–2.2 mm.

***Color.*** Head with frontal surface (Fig. 4C) mostly green to bluish-green, frontovertex variably extensively dark or with slight coppery to reddish-violaceous lusters (Fig. 4A, C). Maxillary and labial palpi dark brown. Antennae with scape mostly yellow, but dorsoapically dark with variably distinct metallic green luster (Fig. 4G); pedicel dark except ventral surface usually paler; flagellum uniformly dark such that mps not conspicuous from surrounding cuticle (Fig. 4G).

Mesosoma (Fig. 4A, B, D) dark with mostly green to bluish-green lusters, though scutellar-axillar complex (Fig. 4A) and dorsellum (Fig. 4E) dark with coppery to reddish-violaceous lusters similar to frontovertex, propodeum (Fig. 4E) more distinctly blue to purple, and mesepisternum posteriorly near metapleuron usually more violaceous (Fig. 4D). Front leg with coxa, trochanter and most of femur dark, but following pale: trochantellus, femur apically, tibia except for dark mesotibial apical pegs, and tarsus except pale often apical tarsomere variably darker brown. Middle leg color pattern similar to front leg except femur often somewhat more extensively pale apically or dorsoapically along anterior margin and apical tarsomere more distinctly dark, but mesotibial spur similarly pale as basal four tarsomeres. Hind leg mostly dark but at least ventral surface of trochantellus, tibia basally for distance less than one-third length of tibia, and basal four tarsomeres pale (Fig. 4B). Fore wing entirely hyaline with yellowish to yellowish-brown venation and dark, hair-like setae.

Gaster mostly dark brown but with variably distinct metallic green luster dorsobasally on Gt1 (Fig. 4B).

***Setation.*** Head with very slightly lanceolate white setae on lower face, interantennal prominence and parascrobal region ventrally, but more hair-like dark setae on parascrobal region dorsally and frontovertex (Fig. 4C), and hair-like white setae on gena. Mesonotum with dark hair-like setae (Fig. 4D), the setae slightly longer and more bristle-like on scutellum. Fore wing (Fig. 4F) cubital cell ventrally with 3–4 rows of setae along entire length and dorsally with line of setae along entire leading margin and at least a partial second row behind first row; basal cell and disk entirely setose except for slender speculum along length of basal fold from parastigma to medial fold, though this variably obscured by setae on ventral surface at least toward parastigma.

***Structure.*** Head in frontal view (Fig. 4C) about 1.2× wider than high, in dorsal view (Fig. 4A) about 2.1× wider than long, and in lateral view (Fig. 4B) about 1.8× higher than long; OOL: LOL: POL: MPOD (allotype) = 1.0: 1.6: 4.0: 1.6. scrobal depression (Fig. 4C) shallow, arch-like, with inclined lateral surfaces extending obscurely to anterior ocellus, but flat surface of depression above toruli usually separated from anterior ocellus by pale, yellowish line almost equal in length to longitudinal diameter of ocellus. Antenna (Fig. 4G) with scape almost 3× as long as maximum width; pedicel subglobular, as long as wide; length of pedicel + flagellum about 2.4× head width; flagellum densely microsetose with numerous rows of densely packed mps (Fig. 4G); fl1 strongly discoidal, not evident between scape and fl2; fl2 dorsoventrally flattened so as to be wider than high and in lateral view distinctly curved with ventral surface concave, but subsequent flagellomeres increasingly subcylindrical and straight (Fig. 4G); length(width) of fl2–fl8 and clava in lateral view (allotype): 24(10), 17(10), 17(10), 14(10), 14(10), 13(10), 10(10), 44(11) (Fig. 4G, H).

Structure of mesosoma and gaster (Fig. 4A–D) typical for the genus. Fore wing (Fig. 4F) 2.2–2.3× as long as wide; SMV: MV: PMV: STV (allotype) = 20: 11: 8.3: 4.7. Protibia with two dorsoapical spicules. Mid leg with spur about as long as basitarsus.

***Sculpture.*** Head with vertex reticulate-rugose to transversely reticulate-strigose; frons and parascrobal regions mesh-like coriaceous to granular, lower face shallowly reticulate to reticulate-imbricate.

Mesoscutum (Fig. 4A) with medial lobe usually more coarsely reticulate-rugulose anteriorly, but at least about posterior two-thirds mesh-like reticulate except sculpture variably extensively finer laterally near notauli and sometimes posteriorly near transscutal articulation, and lateral lobe mostly mesh-like coriaceous; scutellar-axillar complex with dorsal surface of axilla variably distinctly reticulate-rugulose, but larger inclined lateral surface obliquely alutaceous-strigose, and scutellum usually at least slightly reticulate anteromedially but more mesh-like coriaceous to coriaceous-imbricate laterally and posteriorly. Propodeum (Fig. 4E) with complete medial carina; plical region smooth and shiny or at most with extremely obscure mesh-like coriaceous sculpture.

##### Distribution.

*Anastatusgansuensis* (Genbank accession no. MK373759) originally described from China (Gansu Province).

##### Biology.

Solitary endo-parasitoid of the eggs of *C.japonica* in the field and, at least in the laboratory, of *A.pernyi*. *Caligulajaponica* has one generation a year and overwinters in the egg stage; its egg-laying period is from late August to late October in northwestern China (Qiao et al. 2014). In the winter of 2017 were collected a total 283 egg masses of *C.japonica* from Gansu Province and reared them in the laboratory (T: 25±1° C, RH: 70±5%, L: D=14h: 10h). Of these, 12 egg masses (4.2%) were parasitized by *A.gansuensis*. All the emerged wasps of *A.gansuensis* were females. These were subsequently offered the eggs of *A.pernyi* in the laboratory as an alternative host for propagation, which led to the discovery that *A.gansuensis* is thelytokous parthenogenetic. When unmated females were reared on *A.pernyi* eggs in the laboratory both sexes were produced in a highly female-biased sex ratio (sex ratio about 113:1, ♀:♂ = 5987:53; *N* =29). There can be 12–14 generations in one year, with one generation completed in 28–33 days under a 25±1° C temperature and 70±5% humidity regime. The average longevity and fecundity of a female (*N* =29) was 67 (37–93) days and 303 (68–506) eggs, respectively, when fed with honey water (honey: water = 3:7). The results of our rearing experiments indicate that this species has potential as a biological control agent for suppression of *C.japonica*.

##### Etymology.

Derived from the name of the province in China from which the type series was originally collected through rearing.

##### Remarks.

Because of their dark acropleuron, females of *A.gansuensis* key most closely to females of *A.fulloi* in Peng et al. (2017), but the two are readily differentiated by the color, setal and structural features as given in the key and diagnoses. Females key to *A.tenuipes* Bolivar y Pieltain, 1925 in Narendran (2009), in part because the apical margin of the hyaline cross band is angulate and all funiculars are longer than wide in both, but *A.tenuipes* females are readily differentiated by their pale mesosoma. Similarly, depending on the interpretation of whether or not the scrobal depression “by far” does not reach the anterior ocellus, *A.gansuensis* females could key to *A.tenuipes* in Kalina (1981) or to *A.splendens* Nikol’skaya, 1952 and *A.brevicaudus* Kalina, 1981. However, in contrast to the key features of Kalina (1981), the mesoscutum is slightly wider than long (Fig. 2E), unlike that keyed for *A.splendens* and the anterior convex portion of the mesoscutal medial lobe is triangular with gradually convergent sides (Fig. 2E), unlike that keyed for *A.brevicaudus*. Furthermore, females of both *A.brevicaudus* (Kalina 1981, fig. 14) and *A.splendens* have at least the apical two funiculars subquadrate to slightly transverse and the acropleuron pale to coppery.

Two features of the fore wing of female *A.gansuensis* are not apparent in all females: the presence of isolated dark setae within the hyaline cross band medially (Fig. 3C) and a region of more orangish setae behind the parastigma and base of the marginal vein anterior to the medial fold (Fig. 3B). Although the latter feature is distinctive it is not unique to *A.gansuensis*, being possessed also by the brachypterous females of *A.meilingensis* (Fig. 6E) and by some females we recognize as *A.japonicus*, but not by *A.fulloi* females.

**Figure 5.**
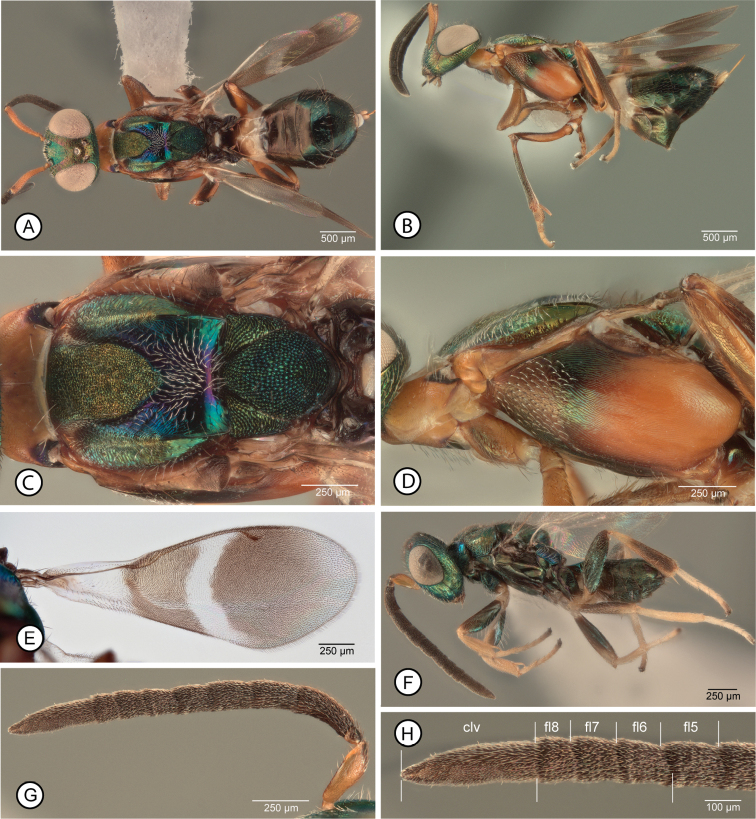
*Anastatusjaponicus***A–E** female: **A** dorsal habitus (6) **B** lateral habitus (4) **C** mesosoma in dorsal view (6) **D** same in lateral view (4) **E** fore wing (5). **F–H** male: **F** lateral habitus (25) **G** antenna (25) **H** clava and apical three funiculars (25) (three lower bars indicate length of clava compared to combined length of apical funiculars). Abbreviations: clv = clava, flx = flagellomere number.

**Figure 6.**
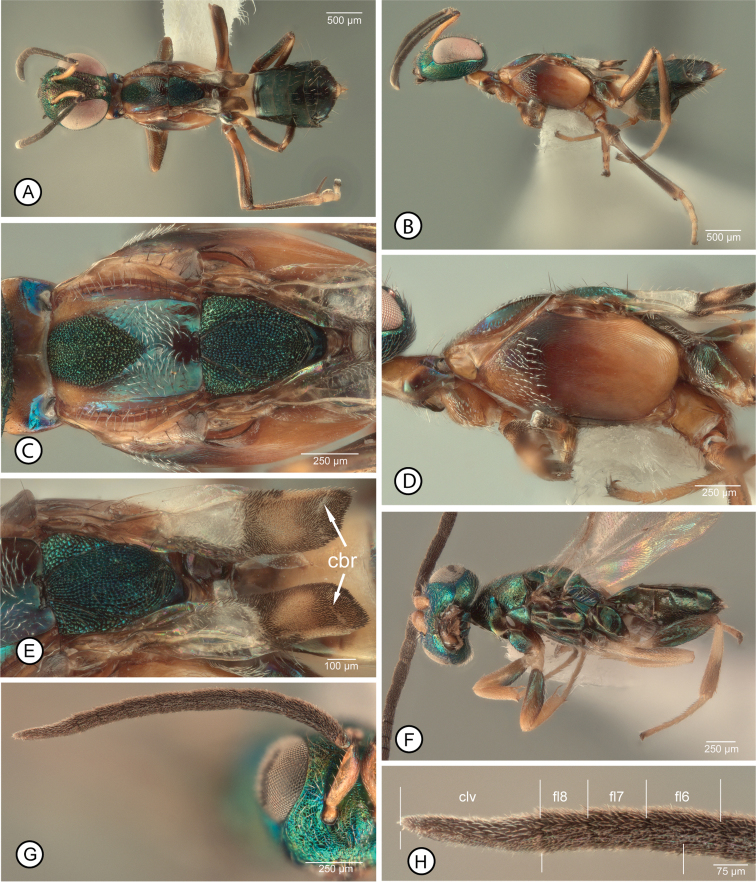
*Anastatusmeilingensis***A–E** female (2): **A** dorsal habitus **B** lateral habitus **C** dorsal mesosoma **D** lateral mesosoma **E** fore wing, dorsal view. **F–H** male (19): **F** lateral habitus **G** antenna **H** clava and apical three funiculars (three lower bars indicate length of clava compared to combined length of apical funiculars). Abbreviations: cbr = remnant of hyaline cross band, clv = clava, flx = flagellomere number.

As noted under *A.fulloi*, a flagellar structure differentiates males of both *A.gansuensis* and *A.meilingensis* from those of *A.fulloi* and *A.japonicus*. Males of the former two species have the clava distinctly shorter than the combined length of the three apical funiculars (Figs 4H, 6H) whereas males of the latter two species have the clava at least as long and sometimes distinctly longer than the combined length of the three apical funiculars (Figs 1H, 5H).

#### Anastatus (Anastatus) japonicus

Taxon classificationAnimaliaHymenopteraEupelmidae

Ashmead

8B90970B-D5BC-5C9C-B024-5F42D12122AB

Fig. 5A–H

Anastatus japonicus Ashmead, 1904: 153; syntypes (USNM), examined.
Anastatus bifasciatus disparis Ruschka, 1921: 265. Synonymy by Bouček 1977: 124–124.
Anastatus disparis ; Burgess 1929: 574, new status for Anastatusbifasciatusdisparis.
Anastatus japonicus ; Yang et al. 2015: 163–164, fig. 84.

##### Diagnosis.

**Female.** Macropterous (Fig. 5A, B); fore wing with broad hyaline cross band behind marginal vein with similarly curved basal and apical margins so band uniformly wide, and without isolated dark setae medially (Fig. 5E). Mesosoma (excluding legs) with mesonotum, prosternum and usually acropleuron anteriorly dark, but at least about posterior two-thirds of acropleuron, pronotum except for dark spot anterior to each spiracle (Fig. 5B, D), prepectus (Fig. 5D), and tegula at least basally (Fig. 5C) contrastingly paler; procoxa, except often in part laterally (Fig. 5D), similarly pale as pronotum and acropleuron posteriorly; mesotarsus with all tarsomeres similarly pale yellowish to white or with basal two tarsomeres only inconspicuously darker infuscate in part (Fig. 5B). Mesoscutum with posterior concave part comparatively broadly setose but white setae not attaining lateral margins (Fig. 5C). Antenna with at least apical funicular slightly transverse and previous one or two funiculars subquadrate.

**Male.** Structure as well as color, setae and sculptural patterns (Fig. 5F) similar to those described for *A.gansuensis* except clava at least about as long as combined length of fl6–fl8 (Fig. 5H), with fl8 and fl7 slightly transverse to quadrate and fl6 and fl5 slightly longer than wide (Fig. 5H).

##### Distribution.

*Anastatusjaponicus* (Genbank accession no. MK604240) was reported previously from China (Beijing, Fujian, Guangdong, Guangxi, Hong Kong, Jiangsu, Jilin, Liaoning, Shaanxi, Shandong) by several authors (Han et al. 1999; He 2001; He et al. 2001). We reared it in the field from the following localities: **Gansu Province**: Kang Co., Longnan City, collected 23.I.2018, Yong-Ming Chen (1♀, 1♂ AICF; 1♀, 1♂ BMNH; 21♀, 16♂ CNC; 5♀, 3♂ FAFU; 7♀,6♂ IZCAS; 1♀, 1♂ USNM). **Jilin Province**: Changchun City, Jilin Agricultural University, 20.VII.2017, Yong-Ming Chen (1♀, 1♂ AICF; 1♀, 1♂ BMNH; 33♀, 17♂ CNC; 1♀, 1♂ USNM).

Extralimital distribution listed for *A.japonicus* by Noyes (2019) includes at least one country from all biogeographic regions except the Neotropical. It is widely distributed throughout the entire Palaearctic region where it is native.

##### Hosts.

Noyes (2019) lists *A.japonicus* as a parasitoid of over 15 host species in two families of Hemiptera (Alydidae, Pentatomidae) and five families of Lepidoptera (Lymantriinae (Erebidae), Lasiocampidae, Notodontidae, Papilionidae, Saturniidae), sometimes as a hyperparasitoid through Braconidae and Encyrtidae primary parasitoids (Peck 1963; Kochetova 1968). It was reared in China previously on *A.pernyi* (Wu et al. 2000) and it has been utilized for biocontrol of the litchi stink bug, *T.papillosa*, being identified in the literature most commonly either as *Anastatus* sp. (Lu and Yang 1983; Lin and Lin 1998) or as *A.japonicus* (Xin and Li 1989, 1990; Tang et al. 1993; Xian et al. 2008; Li et al. 2017). Here we newly report it as an egg parasitoid of *C.japonica*.

##### Remarks.

Females of *A.japonicus* are most easily distinguished from two species with macropterous females reared from *C.japonica* by the acropleuron being extensively paler, lighter brown to yellowish (Fig. 5D), in contrast with its dark mesonotum (Fig. 5C). Females of *A.fulloi* and *A.gansuensis* either have the acropleuron entirely dark (Fig. 1D, 2F), similar in color to the mesonotum, or paler anteriorly only near the base of the prepectus (Fig. 2F).

As noted under *A.fulloi*, males of *A.japonicus* and *A.fulloi* are differentiated from those of *A.gansuensis* and *A.meilingensis* by a longer clava relative to the combined length of the apical funiculars. This is because in *A.fulloi* (Fig. 1H) and *A.japonicus* (Fig. 5H) the apical funiculars are somewhat shorter compared to those of *A.gansuensis* (Fig. 4H) and *A.meilingensis* (Fig. 6H). Currently, we cannot reliably differentiate *A.japonicus* from *A.fulloi* males.

#### Anastatus (Anastatus) meilingensis

Taxon classificationAnimaliaHymenopteraEupelmidae

Sheng

152CC680-1EA4-5FD6-881D-AC5C3CEA16BA

Fig. 6A–H

Anastatus meilingensis Sheng, 1998: 5–6, fig. 1; holotype (JLAU), examined.
Anastaus meilingensis ; Peng et al. 2017: 16–18, figs 34–40.

##### Diagnosis.

**Female.** Brachypterous, fore wing extending only to about level of posterior margin of Gt1 when body uncontorted (Fig. 6A, B); basal 0.6× (basal region) hyaline and much more sparsely setose than densely setose apical 0.4× (discal region) (Fig. 6C); basal region with slightly lanceolate dark setae extending from discal region for half-length within slender vanal region and with white or mostly white hair-like setae immediately anterior to venal fold and at least narrowly along base of discal region, though often bare behind submarginal vein; discal region uniformly covered with slightly lanceolate dark setae except for conspicuous region of orangish setae medially and often variably conspicuous but slender, sometimes interrupted remnant of hyaline cross band apically (Fig. 6: cbr), with orangish setae extending at least to leading margin, including part of marginal vein, though sometimes not to posterior margin of wing so as to be enclosed by dark setae basally and apically and sometimes posteriorly; submarginal vein, including parastigma, extending slightly beyond basal region and partly orangish marginal vein extending most of length of discal region, without differentiated stigmal or postmarginal veins. Mesosoma (excluding legs) with prosternum dark but pronotum mostly pale except for dark spot anterior to each spiracle (Fig. 6C) and posterolateral, vertical surface (panel) often brown to dark (Fig. 6D); prepectus (Fig. 6D) and tegula (Fig. 6C) at least basally pale; mesonotum (Fig. 6C) with scutellar-axillar complex and mesoscutum medially dark, but mesoscutal lateral lobes with at least outer, inclined surfaces partly to mostly pale; acropleuron mostly pale except dark anteriorly in region ventral to tegula (Fig. 6D); procoxa, except often in part laterally, similarly pale as at least anterolateral surface of pronotum and most of acropleuron (Fig. 6D); mesotarsus with all tarsomeres similarly pale yellowish to white (Fig. 6B). Mesoscutum with posterior concave part completely setose with white setae (Fig. 6C). Antenna with at least apical funicular subquadrate and previous two funiculars only slightly longer than wide.

**Male.** Structure as well as color, setae and sculptural patterns (Fig. 6F–H) similar to those described for *A.gansuensis* except about basal half of metatibia pale and apical half lighter brown, lighter in color than respective femur (Fig. 6F).

##### Distribution.

*Anastatusmeilingensis* (Genbank accession no. MK604242) was described originally from Jiangxi Province from three localities (Shangrao, Yushan, and Meiling, Nanchang) as detailed by Peng et al. (2017). We reared *A.fulloi* in the field from the following new locality: **Gansu Province**: Kang Co., Longnan City, 23.I.2018, Yong-Ming Chen (2♀, 2♂ AICF; 2♀, 2♂ BMNH; 58♀, 36♂ CNC; 5♀, 3♂ FAFU; 15♀,12♂ IZCAS; 2♀, 2♂ USNM).

##### Hosts.

Originally described reared from the eggs of the pine moth, *Dendrolimuspunctatus* Walker, and of the Simao pine moth, *D.kikuchii* Matsumura (Lepidoptera, Lasiocampidae) (Sheng and Yu 1988). Here we newly report it as an egg parasitoid of *C.japonica* and, in the laboratory, of *A.pernyi*.

##### Remarks.

Of the species we reared from *C.japonica*, females of *A.meilingensis* are readily distinguished because they are the only ones that are brachypterous. The fore wing color pattern, with the discal region having a distinct region of orangish setae behind the venation and often also a slender remnant of a hyaline cross band more apically (Fig. 6E: cbr), is unique among described *Anastatus* species with brachypterous females. However, except for the reduced hyaline cross band, the setal pattern is similar to that described for *A.gansuensis*. Females of one other described *Anastatus* species from China, those of *A.huangi* Sheng & Yu (1998), are also brachypterous. However, the reduced fore wings of female *A.huangi* have a color pattern more similar to macropterous females, having a distinct hyaline cross band with white, hair-like setae behind the marginal vein (Peng et al. 2017, fig. 32).

Although Sheng and Yu (1998) reared a single male as part of the type series of *A.meilingensis* this male was not found by Peng et al. (2017). The line drawn figure of the antenna stated as that of a male by Sheng and Yu (1998, fig. 4) is obviously that of a female because of the presence of a visible, anelliform fl1, and a three segmented clava. Of the species treated, males of *A.meilingensis* are mostly similar to those of *A.gansuensis* because both have a clava that is distinctly shorter than the combined length of the apical funiculars (Figs 4H, 6H). However, based on our reared material males of *A.meilingensis* have a different metatibial color pattern, the metatibia being more extensively pale basally and lighter brown apically than for males of *A.gansuensis* (cf. Figs 4B, 6F).

## Discussion

Our study newly demonstrates that *Anastatusfulloi*, *A.gansuensis*, *A.meilingensis* and *A.japonicus* are natural egg parasitoids of *Caligulajaponica* and thus are potential biological control agents of this pest in China. Further, all four species were reared successfully on the eggs of the alternate host, *A.pernyi* in the laboratory, as for some *Trichogramma* species (Zhang et al. 2017; Li et al. 2018), which demonstrates the possibility of mass-rearing the species for augmentative release against *C.japonica*. However, further study is required to evaluate their parasitism and possible competition in the field for biological control efficacy, as well as any effects of adaptation on *A.pernyi* eggs upon field-release.

All *Anastatus* species previously reported have been considered to have a haplo-diploid reproductive mechanism, which requires fertilization of the female by a male for female production (Noyes 2019). However, by culturing unfertilized females on *A.pernyi* eggs we discovered that *A.gansuensis* is a thelytokous parthenogenetic species, which implies huge reproductive potential of the female wasps for laboratory experiments and biocontrol. In order to use most effectively a biological control agent within a mass production system, the production of female individuals is clearly desirable because this is the sex responsible for reducing the pest population in the field. It remains to be demonstrated whether thelytokous parthenogenesis is more common in *Anastatus* than for just *A.gansuensis*.

## Supplementary Material

XML Treatment for Anastatus (Anastatus)

XML Treatment for Anastatus (Anastatus) fulloi

XML Treatment for Anastatus (Anastatus) gansuensis

XML Treatment for Anastatus (Anastatus) japonicus

XML Treatment for Anastatus (Anastatus) meilingensis
